# First-Trimester Vaginal Bleeding and Adverse Obstetric Outcomes in a Semi-urban Population of India: A Prospective Observational Study

**DOI:** 10.7759/cureus.64784

**Published:** 2024-07-17

**Authors:** Satish Choudhury, Khushbu Dubey, Bharati Singh Sengar, Surbhi Chetiwar

**Affiliations:** 1 Obstetrics and Gynaecology, All India Institute of Medical Sciences, Bhopal, Bhopal, IND; 2 Obstetrics and Gynaecology, Dr. D. Y. Patil Medical College, Hospital & Research Centre, Pune, IND

**Keywords:** first-trimester bleeding, abortion, preterm labor, pregnancy outcomes, maternal and perinatal outcome

## Abstract

Background

This study aimed to investigate the obstetric outcomes in antenatal women with first-trimester vaginal bleeding.

Methodology

This single-centered, prospective, observational study was conducted in a tertiary healthcare institution. Antenatal women with first-trimester vaginal bleeding who visited the hospital were screened for eligibility and included in the study. They were followed up until the termination of pregnancy or delivery based on the etiology of vaginal bleeding. Various fetomaternal outcomes such as pregnancy outcomes, obstetric complications, mode of delivery, and neonatal outcomes were analyzed.

Results

This study included 120 antenatal women who experienced first-trimester vaginal bleeding during the study period. Vaginal bleeding was more prevalent in the age group of 25-34 years and primigravidas. Out of 120 women, 14 (11.6%) either aborted or the pregnancy was terminated as a result of a nonviable gestation, and 106 (88.4%) delivered after the period of viability. Out of 106 women, 56 (52.8%) had full-term pregnancies without any obstetric complications. We analyzed the obstetric complications developed in all the study participants and found that 23 (21.7%) had preterm labor, 12 (11.3%) had placental abruption, 6 (5.7%) had premature rupture of membrane, 4 (3.9%) had anemia, and 2 (1.9%) developed hypertensive disorder of pregnancy. Of all deliveries, 54 (50.9%) delivered vaginally and 52 (49.1%) had cesarean delivery. There were no major adverse neonatal outcomes in terms of birthweight, APGAR score at one minute, and APGAR score at five minutes.

Conclusions

A large proportion of antenatal women with first-trimester vaginal bleeding can have favorable perinatal outcomes. However, as a few may develop obstetric complications, regular follow-up of such cases is mandated to prevent adverse outcomes.

## Introduction

First-trimester vaginal bleeding is a common symptom of pregnancy, complicating 16-25% of all pregnancies [1,2]. Although there are several predictors of early pregnancy bleeding, its correlation with the obstetric outcome has not been standardized [3]. The outcome of such pregnancies may be affected by the severity of bleeding, gestational age at bleeding, and the cause of bleeding. It is hypothesized that vaginal bleeding in the first trimester may specify an underlying dysfunction of the placenta which may manifest later in pregnancy and can cause unfavorable obstetric outcomes such as preeclampsia (PE), premature rupture of membrane (PROM), preterm labor (PTL), placental abruption (PA), and fetal growth restriction (FGR) [1,4]. Vaginal bleeding in the first trimester is an independent risk factor for adverse obstetric outcomes and its severity may be directly proportional to the amount of bleeding [4,5]. However, the outcome of first-trimester vaginal bleeding is debatable, and it has been estimated that if sonography detects a viable fetus after first-trimester bleeding, 95-98% of pregnancies will continue until 20 weeks gestation [6]. In this study, we analyze the etiology of first-trimester vaginal bleeding and its correlation with obstetric outcomes.

## Materials and methods

In this prospective, observational study, antenatal women presenting with first-trimester vaginal bleeding between October 2019 and September 2021 were screened for recruitment. This study was conducted in the Department of Obstetrics and Gynaecology at a tertiary healthcare institution in India and was approved by the Institutional Ethics Committee, Dr. D. Y. Patil Medical College, Hospital & Research Centre, Pune (approval number: I.E.S.C./240/2019). The primary objective of our study was to evaluate the impact of first-trimester vaginal bleeding on obstetric outcomes in a cohort of pregnant women. The secondary objectives were to compare the prevalence of specific obstetric complications in women with and without first-trimester bleeding to assess the impact of first-trimester bleeding on neonatal outcomes (e.g., birth weight, APGAR scores).

All antenatal women with singleton pregnancy and first-trimester vaginal bleeding were included in the study. Women with chronic hypertension, diabetes mellitus, a history of smoking, thrombophilia, a history of recurrent pregnancy loss, cervical incompetence, local cervical pathology such as erosion or cervical polyp, congenital uterine anomaly, and uterine fibroid were excluded. Patients who provided informed consent for study participation were recruited. The individual patient data were kept confidential.

A thorough history was documented regarding demographic characteristics, parity, gestational age, severity of vaginal bleeding, and any associated symptoms. All study participants underwent a complete general physical and gynecological examination. Relevant hematological, biochemical, and other investigations were sent. Both transabdominal and transvaginal obstetric sonography was performed for evaluation of vaginal bleeding, and a final diagnosis was ascertained based on clinical and sonographic findings. The severity of bleeding was estimated as light or heavy. Bleeding was considered light if its intensity was spotting and heavy if it was similar to the patient’s menstrual bleeding or more.

The patients were either followed up in the antenatal clinic on days 3, 7, and 14 or treated as inpatients based on the severity of bleeding and the necessity of medical interventions such as blood transfusion, management of missed abortion, incomplete abortion, ectopic pregnancy, and molar gestation. In cases of threatened abortion, patients were managed conservatively, and follow-up sonography was performed.

All study participants were followed up until their termination of pregnancy, and various outcome measures were analyzed. The outcome measures were categorized into obstetric outcomes, obstetric complications, mode of delivery, and neonatal outcomes. Obstetric outcomes included first-trimester abortion, second-trimester abortion, PTL, and full-term delivery. Obstetric complications that developed in the antenatal period including PTL, PROM, PA, hypertensive disorders of pregnancy, FGR, and others were analyzed. Neonatal outcomes such as birthweight and APGAR at one and five minutes were studied. The baseline characteristics and outcome measures data were collected from paper-based medical records.

In this study, statistical analysis was conducted to evaluate the relationship between first-trimester vaginal bleeding and various fetomaternal outcomes. Descriptive statistics were used to summarize the demographic data and the prevalence of different obstetric complications. Chi-square tests were applied to compare categorical variables such as the mode of delivery and the occurrence of specific complications. Statistical significance was set at a p-value <0.05.

## Results

A total of 120 patients were recruited in the study and their outcome measures were analyzed. The demographic characteristics, parity, severity, and gestational age at presentation are described in Table 1. Of all study participants, 11.6% of pregnancies resulted in abortion and 88.4% could continue their pregnancy until delivery, among which 19.2% had PTL. The etiology of 14 (11.6%) women with first-trimester vaginal bleeding which resulted in abortion or other procedures because of nonviable pregnancy is depicted in Table 2. The obstetric complications developed were analyzed and showed that out of 120 deliveries, 56 (52.6%) antenatal women had full-term delivery without any obstetric complications. The most frequent obstetric complication was PTL, which accounted for 21.7% of women, followed by PA (11.3%), PROM (5.6%), and anemia (3.9%). Of the 106 women who delivered, 54 (50.9%) had vaginal delivery and 52 (49.1%) were delivered by cesarean delivery. Neonatal outcomes were analyzed as birthweight and APGAR score at one and five minutes (Table 3).

**Table 1:** Demographic and obstetric variables (n = 120).

Variables		n (%)
Age (years)	18–24	34 (28.3)
	25–34	65 (54.2)
	>35	21 (17.5)
Parity	Primigravida	72 (60)
	1	39 (32.5)
	2	6 (5)
	>2	3 (2.5)
Gestational age at bleeding (weeks)	<8 weeks	45 (37.5)
	≥8 weeks	75 (62.5)

**Table 2:** Etiology of abortion/nonviable pregnancy (n = 14).

Etiology	n (%)
Missed abortion	5 (35.7)
Threatened abortion	3 (21.4)
Ectopic pregnancy	3 (21.4)
Incomplete abortion	2 (14.3)
Molar pregnancy	1 (7.2)

**Table 3:** Obstetric and neonatal outcomes.

Outcome		n (%)
Obstetric outcome	First-trimester abortion	9 (7.5)
	Second-trimester abortion	5 (4.1)
	Preterm labor	23 (19.2)
	Full-term pregnancy	83 (69.2)
Obstetric complication	Preterm labor	23 (21.7)
	Placental abruption	12 (11.3)
	Premature rupture of membranes	6 (5.7)
	Anemia	4 (3.9)
	Hypertensive disorder of pregnancy	2 (1.9)
	Intrauterine fetal death	1 (0.9)
	Fetal growth restriction	1 (0.9)
	Rh incompatibility	1 (0.9)
	Full-term pregnancy without any obstetric complication	56 (52.8)
Mode of delivery	Vaginal delivery	54 (50.9)
	Cesarean delivery	52 (49.1)
Birthweight (kg)	<2	8 (7.6)
	<2.1–2.5	21 (20)
	2.6–3	62 (59)
	>3	14 (13.4)
APGAR (one minute)	<5	8 (7.6)
	5–8	79 (75.2)
	>8	18 (17.2)
APGAR (five minutes)	<7	18 (17.2)
	7–9	70 (66.7)
	>9	17 (16.1)

## Discussion

First-trimester vaginal bleeding may be associated with adverse obstetric outcomes apart from higher risk of miscarriages. Bleeding during the first trimester should be evaluated thoroughly as these pregnancies are at high risk of developing PE, PA, FGR, PROM, and PTL [7-9]. It is also associated with low birth weight and poor neonatal outcomes [10]. Invasive trophoblasts and impaired placentation lead to spontaneous miscarriage in early pregnancy while various obstetric complications may result in the second half of pregnancy. Previous studies have documented that there is a robust affiliation between first-trimester per vaginal bleeding and unfavorable fetomaternal outcomes.

In our study, of the 120 cases included, 54.2% were in the age group of 25-34 years, followed by 28.3% in the age group of 18-24 years, and 17.5% in the age group of more than 35 years. Our result suggested that first-trimester vaginal bleeding is more common in primigravida women compared with others. These findings are supported by a few previously conducted studies [11,12]. This could lead the way toward a stronger association of nulliparity with first-trimester vaginal bleeding.

In our study, 83 (69.2%) could continue the pregnancy till full term and PTL occurred in 23 (19.2%) women. The number of first and second-trimester miscarriages was nine (7.5%) and five (4.1%), respectively. A study conducted by Amirkhani et al. concluded that 25% of women had premature labor and 20% of women aborted [11]. Gollapalli et al. concluded that 75.5% of women out of 200 cases reached full term while 11.5% had PTL and the rest aborted either in the first or second trimester [13]. Another study in a tertiary care center in India concluded that out of all antenatal women with first-trimester bleeding, 68.7% delivered at full term whereas 15.3% had PTL and 1.8% aborted in their second trimester [12]. Another study showed that full term was achieved by 78.1% of the total women and 21.9% delivered preterm [14]. Thus, it can be agreed upon that the majority of antenatal women with first-trimester bleeding continue their pregnancy to full term and a few have PTL or abort.

A few women in our study developed obstetric complications such as PA in 11.3%, PROM in 5.7%, anemia in 3.9%, hypertensive disorder in pregnancy in 1.9%, intrauterine fetal death (IUFD) in 0.9%, and FGR in 0.9%. The majority of women (52.8%) with first-trimester bleeding could continue until full term. A study revealed 8.3% of cases with PROM, 13.3% with PA, 1.7% with IUFD, and 0.5% with FGR [11]. Kamble et al. concluded that overall 6.75% of the cases had PROM. In another study, FGR was noted in 14.1% while PROM prevailed in 18.7% and PA was seen in 7.8% of the cases [14]. A study with a large sample size was conducted to compare the incidence of first-trimester bleeding among normotensive versus hypertensive women. It showed that first-trimester vaginal bleeding occurred in 1.6% of cases who later developed PE compared to 2% of normotensives [15]. This suggested that factors associated with PE are inversely proportional to vaginal bleeding in early pregnancy.

While analyzing the mode of delivery, our study showed almost an equal proportion of women had either mode of delivery, i.e., 54 (50.9%) women had a vaginal delivery and 52 (49.1%) had a cesarean delivery. A similar study concluded that 38.4% of cases were delivered by the vaginal route, 41.6% underwent cesarean delivery [11]. A study by Gollapalli et al. showed that among 135 pregnant women who had full-term delivery, 62.22% underwent cesarean section and 37.77% delivered vaginally [13]. However, Kamble et al. concluded that 87 women had a vaginal delivery and 25 women underwent cesarean delivery [12].

In our study, 105 antenatal women delivered live babies, and one was an IUFD case. The maximum proportion of neonates (59%) were in the range of 2.6-3 kg. In the birthweight range of 2.1-2.5 kg, there were 20% of neonates. Overall, 13.4% of cases gave birth to babies weighing more than 3 kg at birth, while 7.6% of cases gave birth to babies weighing less than 2 kg. This finding was supported by a similar study, in which 56.89% of neonates weighed in the range of 2.6-3 kg followed by a weight range of 2.1-2.5 kg which included 18.39% of neonates. Furthermore, it was concluded in this study that 16.09% of neonates weighed more than 3 kg and 8.62% of neonates weighed less than 2 kg.

In our study, 75.2% of neonates had a one-minute APGAR score between 5 and 8. A score of more than 8 was seen in 17.2% while less than 5 was noted in 7.6% of cases. At five minutes after birth, 66.7% of the cases had a score in the range of 7-9. However, the APGAR score of more than 9 was found in 16.1%, while that of less than 7 was noted in 17.2% of cases. There was no neonatal mortality. A similar comparison by Amirkhani et al. concluded that the five-minute APGAR score was less than 7 in 11.7% of the cases [11]. Another study showed that 72.98% of neonates APGAR score of 5-8 at one minute and 66.09% had a score of 7-9 at five minutes after birth. At five minutes after birth, the APGAR score of less than 7 was recorded in nine cases and a score of more than 7 in 151 cases in a study conducted by Kamble et al. [12]. The findings of our study in terms of APGAR score at one and five minutes are consistent with the above-mentioned study.

Congenital anomalies in the offspring born to women who had bleeding in their early pregnancy were not observed in our study. However, a study with a larger sample size needs to be performed, tabulating congenital anomalies with or without bleeding retrospectively or prospectively. Being a prospective, observational study negates the recall bias in our study which can be considered a strength of the study design. A smaller sample size owing to the limited time frame of our study can be considered as the main limitation of our study. This reduced the generalizability of the findings. Due to the smaller sample size in a single center, this study could not be attributed to the association of risk factors with the perinatal outcome. The effects of confounders such as smoking, obesity, socioeconomic factors, and medical disorders on the outcome could not be ruled out in our study. Analyzing the specific causes of first-trimester vaginal bleeding and their impact on fetomaternal outcomes is a critical research priority. Larger multicentric studies addressing the effect of confounders are necessary to confirm the findings of our study.

## Conclusions

Our study observed favorable obstetric outcomes in a significant proportion of women experiencing first-trimester vaginal bleeding. However, the presence of complications in some cases underscores the importance of close clinical monitoring for these women. The limitations of a small sample size and single-center design highlight the need for further research. Future studies with larger, more diverse populations can confirm these findings and explore the association between specific causes of bleeding and obstetric outcomes.
